# Feasibility for Using Thermography Throughout an Exercise Program in Mastectomized Patients

**DOI:** 10.3389/fonc.2022.740787

**Published:** 2022-04-14

**Authors:** Maria Jane das Virgens Aquino, Paula Michele dos Santos Leite, Ingrid Kyelli Lima Rodrigues, Josimari Melo DeSantana

**Affiliations:** ^1^ Physiotherapy Department, Federal University of Sergipe, São Cristóvão, Brazil; ^2^ Physical Therapy Department, Graduate Program in Health Science, Graduate Program in Physiological Science, Federal University of Sergipe, São Cristóvão, Brazil

**Keywords:** breast neoplasm, exercise, feasibility studies, mastectomy, thermography

## Abstract

**Introduction:**

Breast cancer is the most common in the female population. Physical training is safe and indicated after surgical treatment for breast cancer. During exercise, body temperature changes due to tissue metabolic activity; in this sense, infrared thermography is used to map the thermal patterns of the body surface.

**Objective:**

This study aimed to evaluate the feasibility of using thermography during a physical rehabilitation program in mastectomized patients by analyzing the change in body temperature caused by physical exercise in the breast region.

**Methodology:**

This is a simple and covert clinical trial, in which the sample was constituted for convenience. The women were submitted to a supervised physical exercise protocol, three times a week, for 20 sessions. They were evaluated in the first, tenth, and twentieth sessions in relation to changes in body temperature in the breast region (infrared thermography).

**Results:**

Twenty patients who underwent mastectomy surgery were recruited. No patient had drain infection, scar dehiscence, or lymphedema, and only one patient had seroma removed. The mean age was 50.45 ± 2.00 years, and the body mass index (BMI) was 28.95 ± 1.11 kg/m^2^. In the body thermography of the patients’ breast region, no significant difference was observed when comparing the thermograms of the plastron region of the patients in the first, tenth, and twentieth sessions (p = 0.201). However, when comparing the plastron region with the control breast, a reduction in temperature was observed in the operated region in the first (p = 0.012) and tenth sessions (p = 0.004).

**Conclusion:**

Through this study, we can conclude that the use of infrared thermography is viable for the analysis of the body temperature of mastectomized patients during a supervised physical exercise protocol and, therefore, suggest that this instrument is increasingly used in the cancer public.

## Introduction

Breast cancer has the most incidences in both the world and Brazilian female population, except for non-melanoma skin cancer. In Brazil, 66,280 new cases of breast cancer are estimated each year of 2020–2021 biennium (1).

Although physical exercise has a positive impact on the physical and psychological wellbeing of breast cancer survivors, physical activity declines significantly after diagnosis and increases slowly after treatment period (2), and then physical exercise plays a critical role in the recovery. A guideline published by the American College of Sports Medicine (3) states that physical training is safe during and after cancer surgical treatment and improves functionality, quality of life, and oncological fatigue (4).

A systematic review and meta-analysis described the effects of physical exercise interventions after adjuvant therapy in women with breast cancer (5). The main results were improvements in fatigue, cardiorespiratory fitness, quality of life, physical and social/emotional function, and self-reported physical activity, which were sustained for 3 months or more after the intervention. Walking, horse riding, yoga, and water-based and resistance exercises were among the interventions carried out in the studies.

During exercise, body temperature changes due to the metabolic activities of human tissues (6). Thermography is a widely used technique to assess temperature in the tissues because it is a non-invasive and non-painful procedure (7). In oncological patients, this technique can be performed as a complementary examination to detect breast cancer; however, mammography is the primary screening modality (8, 9). Among all pseudo colors, the white region represents a higher temperature indicating an anomalous region of the breast (9, 10). Thermography images offer functional information associated with vasodilatation, hyper-perfusion, and hyper-metabolism (6).

Therefore, infrared thermography (IRT) is a comfortable and safe procedure that aims to capture infrared radiation emitted by a surface to measure different patterns of temperature distribution (11, 12). Besides that, this instrument has been highlighted in terms of its use for rehabilitation purposes, evaluation, and re-evaluation after treatment (9, 13, 14).

IRT is not an instrument that shows anatomic abnormalities, but it is a method that demonstrates physiological changes (15) and allows mapping thermal patterns, that is, thermograms, on body surface (16). Thermograms showing increased nipple temperature, hot spots, and vascular changes may indicate serious breast problems (10). A temperature difference of 1°C between thermograms can be used to detect changes in the breast tissue (17).

A systematic review evaluated skin temperature (T_sk_) during physical exercise in healthy individuals through IRT and observed that there is no homogeneous response in T_sk_ between different regions of the body. The duration and intensity of the proposed activity, the function of the body region, and the need for heat loss are responsible for this heterogeneity (18).

A scientific gap is observed in relation to the clinical safety of therapeutic exercise in post-mastectomized women’s rehabilitation. For a long time, it was recommended that women would remain on bed rest so that they would not have problems with healing or risk of developing metastases. Therefore, this study aims to assess the feasibility of using thermography during a physical rehabilitation program in mastectomized patients by analyzing the change in body temperature promoted by physical exercise in the breast region (postoperative inflammation). Through the use of IT, we can safely conduct the patient’s physical rehabilitation process.

## Material and Method

This is a single-blinded arm of a randomized clinical trial; the sample was obtained by convenience, composed of patients who were looking for treatment at Associação de Amigos da Oncologia (AMO), a non-profit organization in Aracaju, Sergipe, Brazil. Two investigators participated in the study. Investigator 1 performed the initial, middle, and final assessments, and investigator 2 was responsible for supervising the exercise protocol.

The present study was approved by the Committee of Ethics for Research in Humans of the Federal University of Sergipe (2.537.651). Participants signed an informed consent according to Resolution 466 from National Health Council. CONSORT’s recommendations for pilot and feasibility trials were used in the preparation of this study (19).

Women with an anatomopathological diagnosis of breast cancer who underwent either radical or total mastectomy surgery were included. They could have been submitted or not to adjuvant chemotherapy and were admitted at rehabilitation service after drain removal or until 4 months after surgery and with the healed surgical incision. Women with breast reconstruction, metastasis, and bilateral breast cancer or those who presented an orthopedic or rheumatologic problem that represented significant functional alterations (e.g., bursitis, fibromyalgia, and lupus) were excluded.

**Figure 1:**
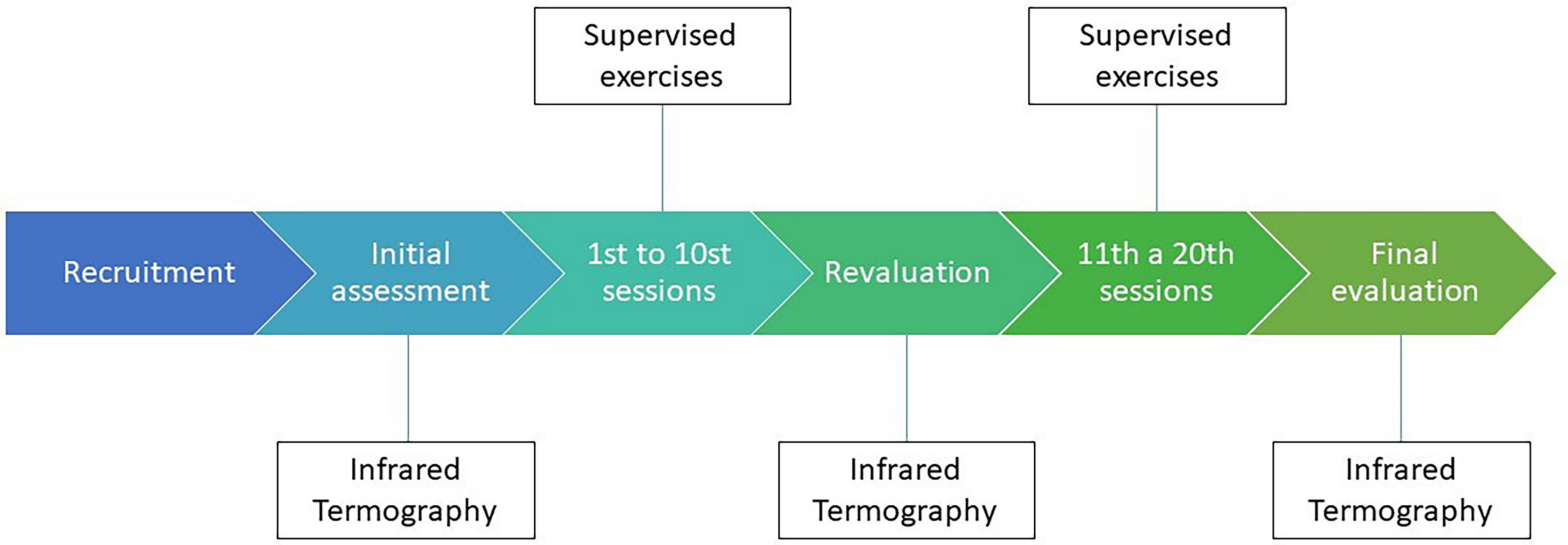
Study timeline.

Women underwent a physical exercise protocol, three times a week, individually, for 50 min, totaling 20 sessions. This quantity of sessions represents the recommended time to achieve improvement in mobility and postoperative pain that may impair the performance of radiotherapy, if indicated, as often used in clinical practice. The format for each exercise session consisted of global stretching, followed by active-free kinesiotherapy, resistance training in the upper and lower limbs, and, finally, a relaxation period with stretching activities (20, 21) (**Figure 1**).

Exercises promoted active shoulder abduction, adduction, flexion, extension, and internal and external rotation movements; elbow flexion and extension; and hip and knee flexion and extension and plantar flexion (20). Three series of 8 to 15 repetitions were done with a resting interval of 1 min, for each exercise. Resistance exercises using 1- and 2-kg dumbbells, 1-kg shin pads, and elastic bands with mild and moderate resistance were inserted as patients presented flexion above 90°, abduction above 45°, and external rotation of the shoulder above 30° in the upper limb ipsilateral to the surgery. In the lower limbs, this therapeutic modality started in the first session.

If some discomfort was perceived by the patient during physical exercises, such as nausea, vomiting, excessive sweating, fatigue, or unbearable pain, exercise was interrupted until the patient felt better. Physical exercises were performed, respecting the limit of each patient involved in the study. The physical therapist responsible for the treatment was trained for it and had 4 years of experience in this clinical area. Patients also received all orientations that should be adopted with the operated region and the ipsilateral arm to the surgery. Likewise, they were encouraged to freely perform aerobic activities, such as walking, dancing, or swimming.

IRT evaluates body abnormalities through alterations in superficial blood flow in affected areas (15). This measurement was performed in the 1st (baseline), 10th (middle), and 20th (final) sessions of the patients, and a thermographic camera FLIR Systems T420™ (Wilsonville, OR, USA) was used as assisted by a computer. This equipment allows images with a spatial resolution of 1.4 mrad obtained by visualization of hot spots of 1.4 mm to 1 m of distance, using standard lenses without additional lenses: thermal sensitivity of 0.05°C in 30°C, a spectral range of 7.5–13 μm, and digital video of 320 × 240 pixels.

All images were analyzed and displayed in a palette of 85–100 colors, with a thermal window of 0.15°C for each color. Thermal sensitivity of 0.51°C per color tone was used based on rainbow type (colorful palette) colorimetric scale, in which the cores were from the warmest to the coldest (FLIR QuickReport™ v. 1.2 and FLIR Reporter™ v. 8.5, FLIR Systems, Inc.). Colors indirectly indicate the degree of distribution of local’s skin cutaneous perfusion.

The room’s temperature was maintained at between 19°C and 21°C (22) and relative humidity of 80%. Women remained in this location, sitting in a chair, with their breasts uncovered for 15 min before images were obtained for acclimatization and to avoid sweating bias. The camera was placed on a tripod at 75 cm in height and 100 cm away from the patient, and the thermograms were captured including both breasts in a single image (11, 14). For the assessment, women were instructed to not apply lotions, makeup, or sunscreen on the skin; not smoke 2 h before evaluation; not drink coffee or alcohol; not perform any physical activity; and inform if they have used any medication. They were also instructed to not palpate, press, and rub the skin at any moment until the thermographic examination was completed (14, 15).

When analyzing the images, a region of interest (IR) was standardized on both breasts of each patient, allowing to calculate the mean and SD of the temperature of that region. IR was defined based on the size of the surgical incision for each patient. The upper limit consisted of the axillary line, and the lower limit was the line drawn at the height of the xiphoid process. The unpaired t-test was used to compare temperatures on the plastron region with the contralateral breast. The repeated-measures ANOVA test was performed to compare temperature alterations during rehabilitation protocol. p < 0.05 value was considered statistically significant.

## Results

Twenty patients who submitted to mastectomy for breast cancer removal were recruited. None of these patients presented drain infection, scar dehiscence, or lymphedema, and only one patient needed seroma removal.

Patients’ mean age was 50.45 ± 2.00 years, and body mass index (BMI) was 28.95 ± 1.11 (kg/m^2^). In general, patients started physical therapy only 38.25 ± 23.13 days after the surgical procedure, 60% underwent a simple mastectomy, 70% had their right breast operated on, 70% were married, 100% used the drain, and 85% underwent neoadjuvant chemotherapy (**Table 1**).

**Table 1:** Sociodemographic, clinical, and surgical data of treated patients (n = 20). .

Variable	Mean ± SD
***Age (years)***	50.45 ± 2.00
***Weight (kg)***	70.52 ± 3.16
***Height (cm)***	1.58 ± 0.01
***Body mass index (kg/m^2^)***	28.95 ± 1.11
***Time for beginning treatment (days)***	38.25 ± 23.13
***Marital status***	**n (%)**
*Single*	3 (15)
*Married*	14 (70)
*Divorced*	1 (5)
*Widow*	2 (10)
***Type of surgery***	**n (%)**
*Simple mastectomy*	12 (60)
*Radical mastectomy*	8 (40)
***Local of surgery***	**n (%)**
*Right*	14 (70)
*Left*	6 (30)
***Neoadjuvant chemotherapy***	**n (%)**
*Yes*	17 (85)
*No*	3 (15)
***Neoadjuvant radiotherapy***	**n (%)**
*Yes*	0
*No*	20 (100)
***Lymphedema***	**n (%)**
*Yes*	0
*No*	20 (100)

Values presented as mean ± SD, and absolute and relative values (%).

In body thermographic examinations of the patients’ breast region, no significant difference was observed when comparing thermograms of patients’ plastron region at the first, tenth, and twentieth sessions (p = 0.201). However, when comparing the plastron region with the control breast, a significant increase in temperature was observed in the operated region at the first (p = 0.012) and tenth sessions (p = 0.004) (**Table 2**).

**Table 2:** Cutaneous temperature in degrees in plastron region and control breast at 1st, 10th, and 20th sessions.

Breast	1st session	p	10th session	p	20th session	p	p (RM)
**Control**	32.91 ± 1.59	0.012^*^	32.82 ± 1.25	0.004^*^	32.36 ± 1.44	0.067	0.201
**Operated**	34.26 ± 1.12		34.17 ± 1.13		33.47 ± 0.90		

Data presented in mean ± SD.

RM, repeated measures.

^*^p ≤ 0.05. Unpaired t-test and ANOVA for repeated-measures test.

**Figure 2:**
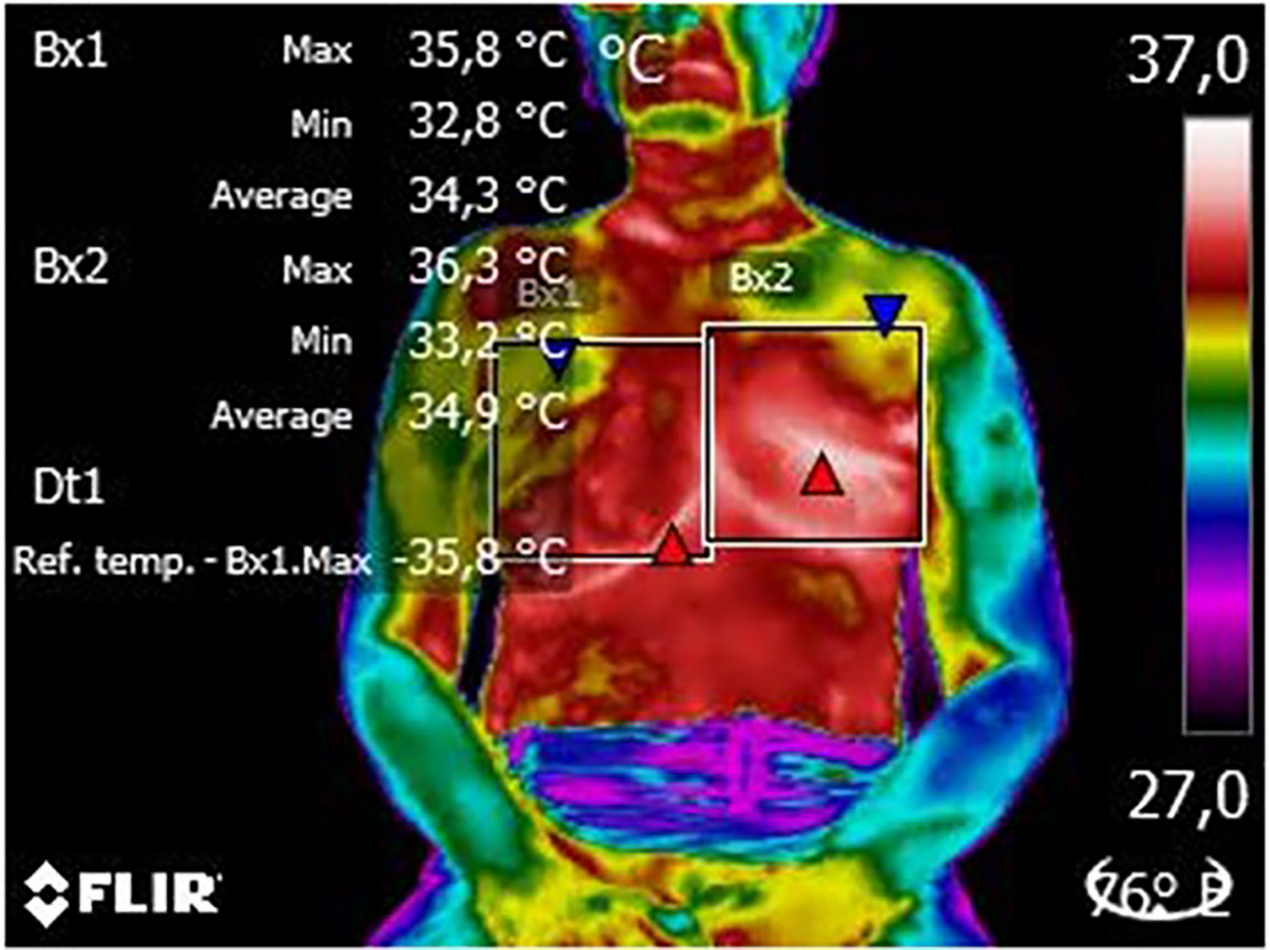
Thermogram of an evaluated patient thermogram. From this image, it is possible to observe the delimitation of the region of interest (white squares), the color palette (frow lowest to highest temperature) used to identify probable changes and the patient’s position (seated) to capture the image. On the right, we can see the patient´s breasts (in red and yellow) while on the left, there is the plastron region (in red) and the mastectomy scar (in white).

In the qualitative analysis of thermograms, we found that all patients (100% of images analyzed) had white and red areas in the region of the surgical scar, which represents higher temperatures at this location, with probable relation to the postoperative inflammatory process (**Figure 2**).

Regarding the feasibility of using thermography in the population studied, no adverse effects were reported by patients; in addition, 100% of participants adhered to the IRT technique and to the proposed physiotherapeutic proposals.

## Discussion

IRT is a non-invasive method that can be performed in any age range, but in the oncologic public, it is mostly used as a complementary exam to cancer clinical diagnosis (7). A study that evaluated the accuracy and reliability of IRT on assessment of breast of women with cancer suggested that it can be used for the purpose of (non-diagnostic) assessment of skin temperature before and after therapeutic procedures or to establish correlations with other clinical variables (17). Biologically, the metabolic rate of cancer cells is higher than that of normal cells. Consequently, tumors act as a heat source, increasing the surface temperatures around the cancerous area, which can be seen as a hot spot in a thermal image (23).

According to the International Academy of Clinical Thermology (IACT), IRT contributes to the evaluation of vascularization in solid organs, diseases in soft tissues, and pain studies (23). In our study, there was an important change in the temperature of the plastron region, which was higher in comparison to that of the control breast. Physical exercise practice, however, caused a reduction in plastron temperature, while the control breast remained unchanged from baseline temperature values. This measurement offers safety and feasibility for a program of supervised rehabilitation after mastectomy. However, body temperature is not the only variable that should be analyzed; the assessment of pain, range of motion, muscle flexibility, and functionality, among others, are also important for the evolution of patients.

After thermographic evaluation of patellar and Achilles tendons of healthy individuals (24), there was an increase in tendon’s temperature immediately after running with an eccentric overload when compared to basal values prior to exercise, between a trained group that used eccentric overload and a non-trained group during 3 days of running. In addition, the study found no difference in temperature between the limbs in the individuals who practiced running. In our study, we can observe that the change in temperature between the breast and the plastron region presents a difference greater than 1°C in the three evaluated moments. This does not mean that the carcinogenesis process is still taking place, as we have to consider the presence of inflammatory mediators in the recent postoperative period. It is noteworthy that the temperature of the plastron region reduces along with each evaluation, which corroborates the hypothesis mentioned above about the influence of the inflammatory cascade.

Fernandes and collaborators (12) monitored changes in the skin temperature of healthy individuals during exercise in 28 body regions of interest (forehead, face, chest, abdomen, back, neck, lumbar spine, hands, forearms, arms, thighs, and legs) and observed a reduction in temperature in most regions of interest after 10 min of activity, with the exception of the lower limbs. However, after an hour of recovery, in the anterior view of the hands and thighs and the posterior view of the legs, there was a significant increase in temperature in relation to the pre-exercise. Nevertheless, in mastectomized women, we noticed that the temperature reduction in the plastron region was maintained after physical exercise protocol, which consisted of 20 supervised training sessions. Furthermore, there was no significant change in body temperature that represents a risk or contraindication to exercise because it promotes the process of carcinogenesis or increases local inflammation.

The application of IRT requires some important care to avoid biases during the acquisition of thermograms. One of them is the regulation of body temperature. Our patients remained for a certain time in acclimatization in a temperature-controlled room and received guidance on the care they should adopt before performing IRT. Thus, sweating would not become a confounding factor at the time of data interpretation. Studies show that the evaporation of sweat that occurs during exercise corresponds to a physiological mechanism to reduce skin temperature (T_sk_) (18, 25). In a thermoneutral environment and at rest, T_sk_ tends to remain in balance, and the change in it may be indicative of the presence of some pathological state (26).

The limitations of our study include the difficulty in segmenting the breast area, since each person has different anatomy, given the amorphous nature of breast structure (10) and the small sample size (n = 20). However, it is important to highlight the innovative character of our study, since there is no article that has assessed the change in body temperature during and after a supervised program of exercises for mastectomized women. Another gap in the literature concerns the lack of studies that address physical therapy treatments and the use of IRT, especially aimed at the breast region. Through this measure, we can show the feasibility of using IRT throughout an exercise program in mastectomized patients by bringing safety to perform physical rehabilitation from a clinical and physiological point of view.

## Conclusion

We can conclude that the use of IRT is feasible for the analysis of the body temperature of mastectomized patients during and after a supervised physical exercise protocol. Thus, we reinforce the possibility for considering this instrument to be increasingly used for evaluating oncology patients.

## Data Availability Statement

The raw data supporting the conclusions of this article will be made available by the authors, without undue reservation.

## Ethics Statement

The studies involving human participants were reviewed and approved by the Committee of Ethics for Research in Humans of the Federal University of Sergipe (2.537.651). The patients/participants provided their written informed consent to participate in this study. Written informed consent was obtained from the individual(s) for the publication of any potentially identifiable images or data included in this article.

## Author Contributions

MA: first authorship. PL: equal contribution. IR: equal contribution. JDS: senior authorship. All authors listed have made a substantial, direct, and intellectual contribution to the work and approved it for publication.

## Conflict of Interest

The authors declare that the research was conducted in the absence of any commercial or financial relationships that could be construed as a potential conflict of interest.

## Publisher’s Note

All claims expressed in this article are solely those of the authors and do not necessarily represent those of their affiliated organizations, or those of the publisher, the editors and the reviewers. Any product that may be evaluated in this article, or claim that may be made by its manufacturer, is not guaranteed or endorsed by the publisher.
